# A SAGE based approach to human glomerular endothelium: defining the transcriptome, finding a novel molecule and highlighting endothelial diversity

**DOI:** 10.1186/1471-2164-15-725

**Published:** 2014-08-27

**Authors:** Guerkan Sengoelge, Wolfgang Winnicki, Anne Kupczok, Arndt von Haeseler, Michael Schuster, Walter Pfaller, Paul Jennings, Ansgar Weltermann, Sophia Blake, Gere Sunder-Plassmann

**Affiliations:** Department of Medicine III, Division of Nephrology and Dialysis, Medical University of Vienna, Waehringer Guertel 18 – 20, A-1090 Vienna, Austria; Center for Integrative Bioinformatics Vienna, Max F. Perutz Laboratories, Medical University of Vienna/University of Vienna, Dr. Bohr-Gasse 9, A-1030 Vienna, Austria; EMBL, European Bioinformatics Institute, Wellcome Trust Genome Campus, Hinxton, Cambridgeshire, CB10 1SD UK; Department of Physiology, Medical University of Innsbruck, Christoph-Probst-Platz 1, Innrain 52, A-6020 Innsbruck, Austria; Department of Haematology and Oncology, Elisabethinen Hospital, Fadingerstraße 1, A-4020 Linz, Austria; London Research Institute, Lincoln’s Inn Fields Laboratories, Cancer Research UK, 44 Lincoln’s Inn Fields, London, WC2A 3LY UK

**Keywords:** Bioinformatics, Endothelial diversity, Glomerular endothelial cell, Neurogranin, Serial analysis of gene expression

## Abstract

**Background:**

Large scale transcript analysis of human glomerular microvascular endothelial cells (HGMEC) has never been accomplished. We designed this study to define the transcriptome of HGMEC and facilitate a better characterization of these endothelial cells with unique features. Serial analysis of gene expression (SAGE) was used for its unbiased approach to quantitative acquisition of transcripts.

**Results:**

We generated a HGMEC SAGE library consisting of 68,987 transcript tags. Then taking advantage of large public databases and advanced bioinformatics we compared the HGMEC SAGE library with a SAGE library of non-cultured *ex vivo* human glomeruli (44,334 tags) which contained endothelial cells. The 823 tags common to both which would have the potential to be expressed *in vivo* were subsequently checked against 822,008 tags from 16 non-glomerular endothelial SAGE libraries. This resulted in 268 transcript tags differentially overexpressed in HGMEC compared to non-glomerular endothelia. These tags were filtered using a set of criteria: never before shown in kidney or any type of endothelial cell, absent in all nephron regions except the glomerulus, more highly expressed than statistically expected in HGMEC. Neurogranin, a direct target of thyroid hormone action which had been thought to be brain specific and never shown in endothelial cells before, fulfilled these criteria. Its expression in glomerular endothelium *in vitro* and *in vivo* was then verified by real-time-PCR, sequencing and immunohistochemistry.

**Conclusions:**

Our results represent an extensive molecular characterization of HGMEC beyond a mere database, underline the endothelial heterogeneity, and propose neurogranin as a potential link in the kidney-thyroid axis.

**Electronic supplementary material:**

The online version of this article (doi:10.1186/1471-2164-15-725) contains supplementary material, which is available to authorized users.

## Background

Endothelial cells (EC) are frequently thought to be homogenous because of the multiple functions they share independent of the organ they serve, such as providing a non-thrombogenic surface, regulation of production or inhibition of vasoactive substances, haemostasis as well as leukocyte recruitment. Yet on closer examination, they show significant heterogeneity between similar vessels in different organ systems or in arterial versus venous endothelia [1–5]. Although known as highly specialized cells since the description of their fenestrated phenotype by F. Jorgensen more than 40 years ago [6] glomerular endothelial characteristics remain largely undefined.

Global gene expression studies added large amounts of valuable information to our knowledge on various EC and related pathologies, e.g. atherosclerosis [7]. Yet, in the case of human glomerular microvascular endothelial cells (HGMEC) developments did not have comparable pace. Due to challenges in obtaining, culturing and maintaining HGMEC studies employing primary cells in human glomerular research have been scarce, but very useful in obtaining new insights of human glomerular endothelium; most recently Amaral et al. investigated how Shiga toxin type-2 and Subtilase cytotoxin lead to damages characteristic for haemolytic uremic syndrome [8]. The ultimate aim to experiment with non-cultured glomerular EC has never been attained and the very first human glomerular endothelial cell line (GEnC) was not presented until 2006 [9]. This cell line has proved to be a useful tool in kidney research. Recently, glomerular endothelial barrier function and its regulation were finally studied in great detail using this tool while previously filtration barrier function research has mostly been on podocytes and the contribution of the glomerular endothelium had been relatively neglected [10]. These studies showed how reactive oxygen species present in common pathologies such as diabetes cause glomerular injury by directly disrupting glycocalyx and how chondroitin sulphate controlled by vascular growth factors A and C contributes to glomerular endothelial glycocalyx modulating the protein passage [8, 11]. Nevertheless, despite its usefulness in endothelial research an immortalized cell line is not suitable to define the cellular transcriptome with its predominant and specific transcripts. Thus, we hypothesized that such an investigation using genetically unmodified HGMEC would enhance our understanding regarding the source of the unique morphological characteristics, the behaviour in both culture and in disease and prepare the grounds for further studies of HGMEC. We used serial analysis of gene expression (SAGE), because it provides an unbiased approach to gene discovery and enables quantitative acquisition of most transcripts expressed [12]. Secondly, SAGE has become a powerful tool due to creation of large datasets holding more than two hundred million tags from a wide spectrum of tissues or cells including different EC which are publicly available as part of the Cancer Genome Anatomy Project [13].

The goals of this study were to establish extensive transcriptomic data as a step towards identification of the transcripts controlling the distinctive morphological and functional characteristics of glomerular endothelium and to underline endothelial diversity by comparisons between SAGE libraries from glomerular endothelial and uncultured glomeruli or from other non-glomerular EC. To reach these goals based on the known challenge in receiving useful data out of large transcript lists we describe a research strategy powerful enough to first confirm the endothelial origin of the transcript lists by bioinformatics, then to identify a low abundant transcript, neurogranin (NRGN) for the first time in glomerular endothelium using an *in-silico* analysis and finally to verify its expression *in vitro* and *in vivo* by means of sequencing and immunohistochemistry.

## Results

### Characterisation of HGMEC

**Figure 1:**
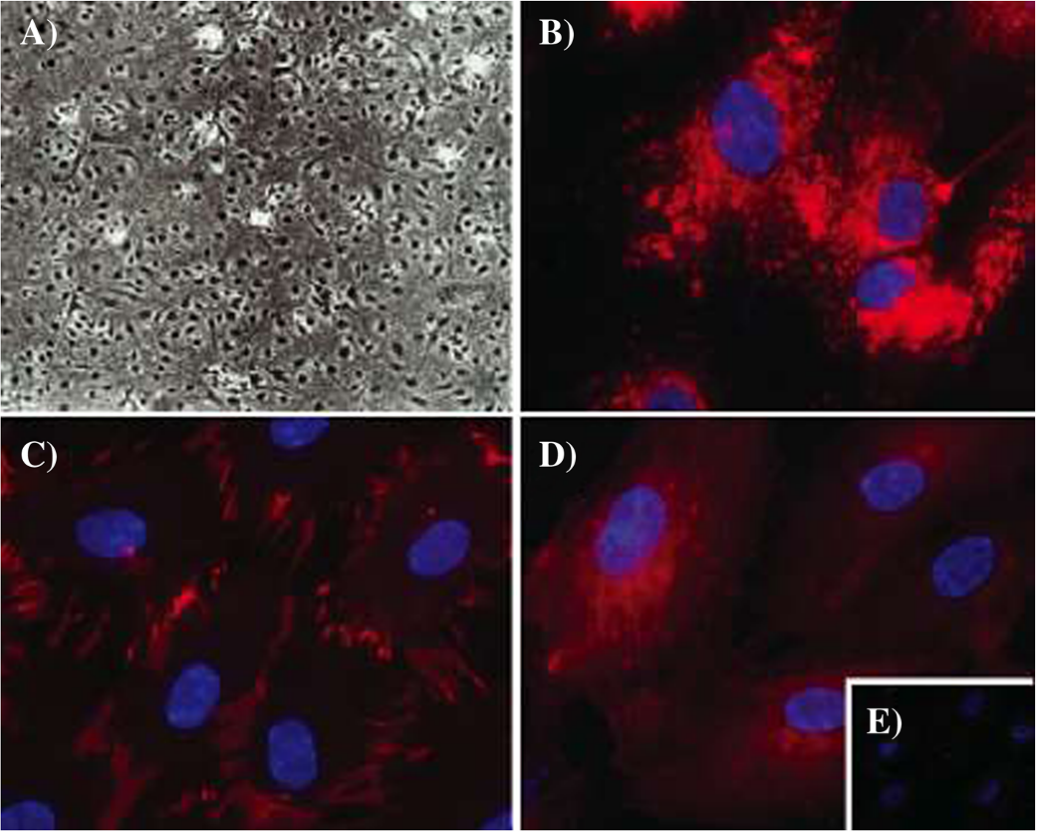
**Characterisation of cultured human glomerular microvascular endothelial cells (HGMEC). A)** Phase contrast micrograph of passage 3 purified HGMEC (magnification 200x), **B** and **C)** Immunofluorescence images of HGMEC probed for von Willebrand factor and PECAM1, respectively. **D)** and **E)** E selectin: HGMEC in panel **D)** were incubated for 12 hours with TNF alpha prior to fixation. No staining with E selectin was observed in unstimulated cells **(E)**. Texas red conjugated secondary antibodies were used for detection and nuclei were counter stained with Hoechst dye (blue). Original magnification in B-E was 630x.

Primary HGMEC formed monolayers and displayed typical cobblestone morphology (Figure 1A) in phase contrast microscopy. Immunofluorescence studies revealed distinct expression of von Willebrand Factor (vWF) and platelet/endothelial cell adhesion molecule 1 (PECAM1, CD31). Von Willebrand Factor staining demonstrated discrete, granular, perinuclear localisation (Figure 1B), whilst CD31 was expressed at the region of cell-to-cell contacts (Figure 1C). HGMEC retained functional characteristics of the microvasculature, expressing E-selectin and P-selectin (CD62E/P) in response to tumor necrosis factor (TNF) stimulation (Figure 1D), whereas unstimulated cells did not (Figure 1E).

**Figure 2:**
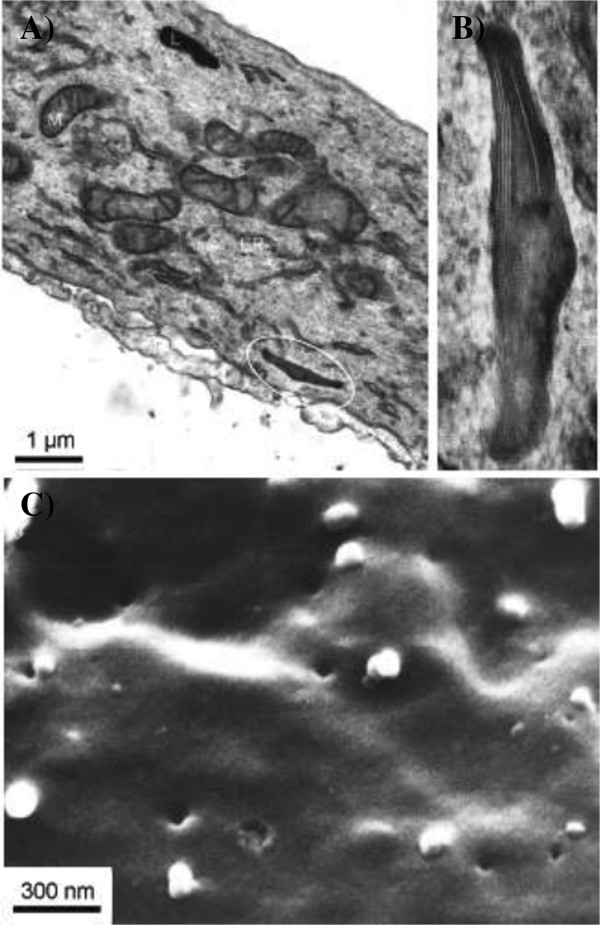
**Electron microscopy (EM) of cultured human glomerular microvascular endothelial cells (HGMEC). A)** HGMEC were cultured on nitrocellulose membranes and processed for EM. Transmission EM showing general cell structures such as lysosome (L), mitochondria (M), and endoplasmic reticulum (ER). Also the endothelial characteristic Weibel Palade bodies can be seen (circle). **B)** Magnification of a Weibel Palade body from panel **A)**, showing rod shaped microtubules. **C)** Scanning EM of a HGMEC cell showing numerous fenestrae.

Transmission electron microscopy showed the presence of rod shaped microtubulated Weibel-Palade bodies (Figure 2A and B) which unambiguously identify the cells as endothelial [14]. Scanning electron microscopy demonstrated numerous fenestrae with a diameter of approx. 100 nm (Figure 2C). The presence of fenestrae as a hallmark of glomerular endothelial reflects the well-differentiated status of these cells [15].

### HGMEC SAGE library

The final HGMEC SAGE library which was constructed using the short SAGE protocol as it is superior to the long SAGE protocol in identifying differential expression of tags [16] contained 68,987 tags with 18,385 unique tags after electronic removal of contaminating linker sequences (Additional file 1: Table S1). It has been approved by the Gene Expression Omnibus (GEO) data depository (http://www.ncbi.nlm.nih.gov/geo) and assigned an accession number [GEO:GSM16892]. Key features of this library are shown in Table 1.

**Table 1:** **Key features of the HGMEC SAGE library**

Frequency	Genes	% of genes	Tags	% of tags	# of reliable unique tags	# of unique tags with no match
**> 10**	1,038	5.6	39,078	56.6	908	3
**5-9**	1,163	6.3	7,541	10.9	938	14
**2-4**	4,019	21.9	10,203	14.9	2,465	170
**1**	12,165	66.2	12,165	17.6	3,239	1,608
**Total**	18,385		68,987		7,550	1,795

Distribution of tags and genes (unique tags) based on SageGenie data.

Reliable tags: All tags with a ranking according to SAGE Genie [13] greater than 80% and not from an undefined 3’ end. Tags with no match: All tags with no matching tags in the SAGE Genie database.

### Verification of endothelial origin of HGMEC SAGE library

**Figure 3:**
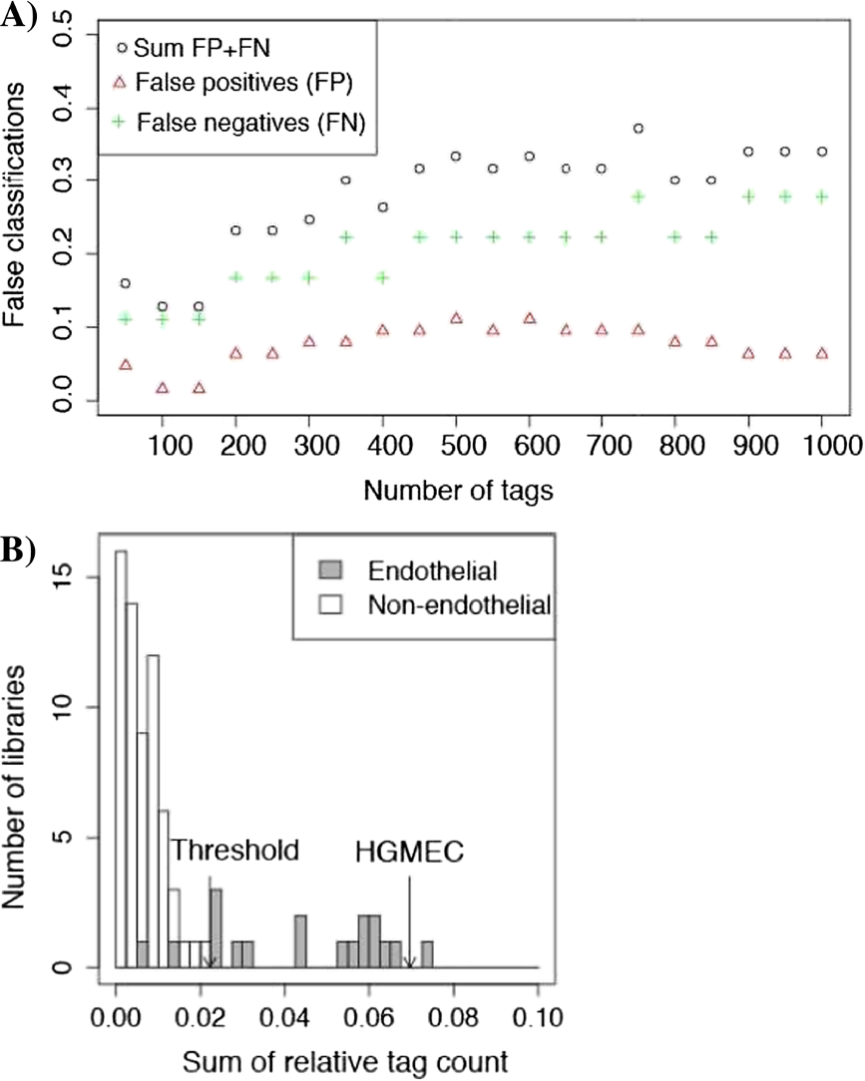
**Classification of glomerular microvascular endothelial cell (HGMEC) library as endothelial. A)** Cross-validation using 63 non-endothelial and 18 endothelial SAGE-libraries not including HGMEC SAGE library. The minimum number of false classifications (false positive: a non-endothelial library classified as endothelial and false negative: an endothelial library classified as non-endothelial) was observed for n = 100 or 150 tags. **B)** Histogram showing the sum of relative expression for each of the endothelial and non-endothelial SAGE libraries as used for cross-validation in panel **A)**. The sum of relative tag counts of 150 tags discriminates between endothelial and non-endothelial SAGE libraries. If the sum is larger than the threshold 0.022, the library is characterised as endothelial. This threshold is clearly exceeded by the HGMEC SAGE library (0.07).

The library was confirmed to be of endothelial origin with a classification approach as explained in detail in the Methods section. In short, we used the sum of the relative expression of 150 tags (Additional file 2: Table S2) as a test statistic: a value larger than the threshold 0.022 indicates an endothelial origin. In other words, if the sum of the total copy numbers of these tags account for 2.2% or more, that library qualifies as endothelial. With a sum of 0.070 (7%) for these 150 tags our HGMEC SAGE library is clearly classified to be of endothelial origin (Figure 3). Notably, two of the analyzed 18 endothelial cell SAGE libraries, “Vascular_endothelium_normal_breast_associated_P1H12 + _AP_1” [GEO: GSM384017] and “Normal corneal endothelium” [GEO: GSM1652], scored below the threshold of 0.022. Consequently, although this might be the reflection of a significant endothelial heterogeneity, only the remaining 16 endothelial cell SAGE libraries were merged to build a non-glomerular endothelial reference SAGE library for further analyses.

### Overlap analysis and mapping

**Figure 4:**
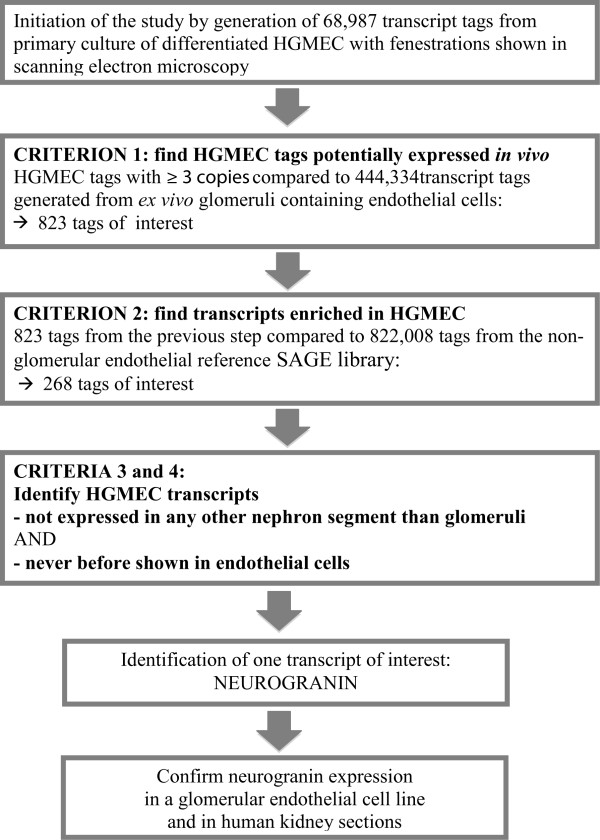
**Short explanation of research strategy: definition of HGMEC transcriptome by generation of SAGE library and identification of HGMEC predominant transcripts based on 4 criteria.** HGMEC: human glomerular microvascular endothelial cell.

After verification of the endothelial origin of this library, we took advantage of the existing public data (SAGE Genie) to reduce the high number of tags to a small group of relevant and differentially expressed tags in HGMEC. The algorithm we used as the research strategy is depicted in Figure 4. First, we determined overlapping transcripts from both the HGMEC SAGE library and the *ex vivo* glomerular library containing 44,334 tags [17]. This showed that 823 transcripts were shared by the cultured HGMEC and the non-cultured glomeruli that contain EC. This group represented transcripts in our HGMEC SAGE library with the potential of *in vivo* expression. Out of these 823 transcripts (Additional file 3: Table S3) 268 were differentially overexpressed in HGMEC using Chi-square test with p < 0.01 in comparison to the non-glomerular endothelial reference SAGE library (822,008 tags) and with the restriction that the observed count in HGMEC is higher than statistically expected (see Additional file 4: Table S4 for the complete list and Table 2 for the 50 most abundant tags as well as Additional file 5: Figure S1 for expression pattern by means of a volcano plot). To test whether our statistical analysis settings were adequate we applied multiple test correction to all p-values of the above described 823 transcripts by means of “false discovery rate (FDR)”. This p-value cut-off only included tags with a false discovery rate of < 0.05 (Additional file 4: Table S4) and was thus expected to result in less than 5% false positives. Surprisingly, this procedure led to the detection of five additional overexpressed transcripts and therefore was less stringent than our previous p-value cut-off of 0.01 with no correction (Additional file 3: Table S3, the additional five transcripts are labelled). Adding these transcripts to the set of overexpressed transcripts did not have an influence on our data or their interpretation. Nevertheless, we added the FDR corrected p-values to Table 2, Additional file 3: Table S3 and Additional file 4: Table S4.

**Table 2:** **Expression of the 50 most abundant from 268 tags which were common to HGMEC and** ***ex vivo*** **glomerular SAGE libraries and overexpressed in HGMEC in comparison to the cumulative endothelial cell library**

UniGene	P-value	FDR corrected p-value	Endo	HGMEC	Expected HGMEC	Description	Confirmed by RT-PCR
hs.425125	0.00E + 000	0.00E + 000	145	551	88.38	Ribosomal protein L29 (Hs00988959_gH)	+
hs.514581	0.00E + 000	0.00E + 000	103	482	74.29	Actin, gamma 1	
hs.586423	0.00E + 000	0.00E + 000	29	434	58.80	Eukaryotic translation elongation factor 1 alpha 1 (Hs00265885_g1)	+
hs.182825	0.00E + 000	0.00E + 000	83	413	62.99	Ribosomal protein L35	
hs.632703	0.00E + 000	0.00E + 000	8	407	52.70	Ribosomal protein L41	
hs.445351	0.00E + 000	0.00E + 000	33	394	54.22	Lectin, galactoside-binding, soluble, 1 (galectin 1)	
hs.522584	0.00E + 000	0.00E + 000	18	380	50.54	Thymosin beta 4, X-linked (Hs03406519_gH)	+
hs.374596	0.00E + 000	0.00E + 000	53	353	51.56	Tumor protein, translationally-controlled 1	
hs.91481	0.00E + 000	0.00E + 000	24	319	43.56	EGF-like-domain, multiple 7 (Hs00211952_m1)	+
hs.523185	0.00E + 000	0.00E + 000	62	286	44.19	Ribosomal protein L13a	
hs.524910	0.00E + 000	0.00E + 000	32	252	36.06	Ferritin, heavy polypeptide 1	
hs.546286	0.00E + 000	0.00E + 000	23	237	33.02	Ribosomal protein S3 (Hs02385124_g1)	+
hs.265174	1.96E-305	1.24E-303	9	215	28.45	Ribosomal protein L32	
hs.494691	1.20E-288	7.05E-287	199	355	70.35	Profilin 1 (Hs00277097_m1)	+
hs.446574	4.37E-285	2.40E-283	13	206	27.81	Thymosin beta 10 (Hs00363670_m1)	+
hs.400295	4.05E-268	2.08E-266	0	179	22.73	Ribosomal protein L30	
hs.388664	8.04E-253	3.89E-251	49	220	34.16	Ribosomal protein L11	
hs.546269	4.36E-246	1.99E-244	130	277	51.68	Ribosomal protein L10a	
hs.433427	1.91E-226	8.27E-225	45	198	30.86	Ribosomal protein S17	
hs.515070	1.04E-224	4.28E-223	28	181	26.54	Eukaryotic translation elongation factor 2	
hs.144835	4.47E-213	1.75E-211	66	206	34.54	Eukaryotic translation elongation factor 1 gamma	
hs.523463	9.00E-212	3.37E-210	50	192	30.73	Ribosomal protein L27a (Hs00741143_s1)	+
hs.275243	9.12E-204	3.26E-202	0	136	17.27	S100 calcium binding protein A6 (Hs01002197_g1)	+
hs.654404	1.62E-196	5.56E-195	182	269	57.27	Major histocompatibility complex, class I, C	
hs.437594	1.55E-187	5.10E-186	79	197	35.05	Ribosomal protein, large, P2	
hs.397609	1.12E-182	3.55E-181	12	136	18.79	Ribosomal protein S16	
hs.111779	1.18E-178	3.60E-177	32	153	23.49	Secreted protein, acidic, cysteine-rich (osteonectin) (Hs00234160_m1)	+
hs.438429	1.19E-170	3.50E-169	177	244	53.46	Ribosomal protein S19	
hs.605502	4.11E-150	1.17E-148	12	114	16.0	Heat shock 70 kDa protein 5 (glucose-regulated protein, 78 kDa) (Hs00607129_gH)	+
hs.546288	8.08E-149	2.22E-147	144	207	44.57	Ribosomal protein S9	
hs.632717	1.24E-145	3.29E-144	88	171	32.89	Myosin, light chain 6, alkali, smooth muscle and non-muscle (Hs02597812_g1)	+
hs.627414	3.86E-142	9.93E-141	102	177	35.43	Ribosomal protein S18	
hs.381219	1.80E-138	4.49E-137	10	104	14.48	Ribosomal protein L15	
hs.410817	7.03E-122	1.70E-120	200	211	52.19	Ribosomal protein L13	
hs.511605	3.74E-121	8.79E-120	21	103	15.75	Annexin A2 (Hs00743063_s1)	+
hs.17441	6.13E-121	1.40E-119	28	109	17.40	Collagen, type IV, alpha 1 (Hs00266237_m1)	+
hs.520898	9.10E-117	2.02E-115	6	85	11.56	Cathepsin B (Hs00947433_m1)	+
hs.356572	2,11E-115	2,11E-115	63	132	24,76	Ribosomal protein S3a	
hs.170622	1.25E-112	2.64E-111	378	275	82.92	Cofilin 1 (non-muscle) (Hs02621564_g1)	+
hs.278573	2.63E-112	5.41E-111	6	82	11.17	CD59 molecule, complement regulatory protein (Hs00174141_m1)	+
hs.433701	1.37E-109	2.75E-108	126	163	36.70	Ribosomal protein L37a	
hs.8372	2.40E-104	4.70E-103	23	93	14.73	Ubiquinol-cytochrome c reductase, 6.4 kDa subunit	
hs.527193	9.87E-103	1.89E-101	22	91	14.35	Ribosomal protein S23	
hs.387208	8.69E-096	1.63E-094	13	78	11.56	Finkel-Biskis-Reilly murine sarcoma virus (FBR-MuSV) ubiquitously expressed	
hs.437191	1.83E-094	3.35E-093	23	86	13.84	Polymerase I and transcript release factor (Hs00396859_m1)	+
hs.591346	6.61E-094	1.18E-092	63	114	22.48	Connective tissue growth factor (Hs01026926_g1)	+
hs.644628	1.40E-091	2.45E-090	167	166	42.29	Ribosomal protein L4	
hs.77961	2.67E-089	4.58E-088	44	98	18.03	Major histocompatibility complex, class I, B	
hs.594444	2.09E-088	3.51E-087	8	68	9.65	Lamin A/C (Hs00153462_m1)	+
hs.446628	4.24E-087	6.98E-086	81	119	25.40	Ribosomal protein S4, X-linked	

UniGene numbers represent the UniGene groups to which the tags are assigned. **P-value** refers to expression comparison between HGMEC and Endo. **FDR:** multiple test correction using false discovery rate. **Endo:** Detected total copy number in the non-glomerular endothelial reference SAGE library. **HGMEC:** Detected copy number in HGMEC. **Expected HGMEC:** number of this particular tag statistically expected in HGMEC SAGE library by means of Chi-square statistics; **Confirmed by RT-PCR:** for these randomly chosen tags (n = 20) qRT-PCR was performed to confirm their expression in HGMEC. **+:** confirmation by qRT-PCR was successful (thus expression of all 20 transcripts confirmed by a TaqMan® Assay; each assay ID is shown in brackets).

The expression of 20 randomly chosen tags from Table 2 was confirmed by quantitative RT-PCR (qRT-PCR). The cytogenetic locations of the 268 transcripts are noted in Additional file 6: Figure S2. According to this analysis chromosomes 1, 6, 11, 17 and 19 are carrying 43% of these genes.

### Gene Ontology (GO) analysis

We identified the GO terms present in the 268 overexpressed genes by mapping them to the GO Biological Process and determining whether they occur more often in a category than expected. We defined a category to be redundant if its child term contained the same genes. Among the 60 detected categories 12 redundant child terms were deleted. The remaining most significant categories resulted in five connected components in the Gene Ontology graph that are listed in Table 3. The known interplay between different ubiquitination processes and apoptosis in different biological systems such as TNF receptor signaling [18] is reflected in HGMEC by the overexpression of many GO terms from these biological processes. Ubiquinol to cytochrome c (“Translation and energy metabolism” cluster) and nuclear migration (“Nuclear migration” cluster) are not displayed in Table 3 despite their significant overexpression due to the small numbers of molecules in the GO-terms mitochondrial electron transport. Nonetheless, in the case of “nuclear migration” (thought to be a common process of neuroepithelia in development and enabling the migration of nucleus between apical and basal surfaces) both of the molecules in this cluster were overexpressed in HGMEC and also in the regulatory microtubule associated molecule Tpx2 [19]. This is not a high abundance molecule and yet it was clearly detected in our HGMEC SAGE library (5.8 copies/200,000). Our GO analysis demonstrates that HGMEC have high expression of ribosomal proteins (23 out of 50 most abundant tags, Table 2). Also, molecules involved in interspecies interactions and cytoskeleton organization are enriched in HGMEC. One of two other molecules of interest in the glomerular endothelium, the von Willebrand cleaving protease ADAMTS13 [GenBank: NM_139025], tag CAGGCTGAAA, was not detected in HGMEC or in any other endothelial SAGE library except in the endothelium of the normal colon (12 copies/200,000). On the other hand caveolin-1 involved in endo- and transcytosis [20] (CAV1, [GenBank:NM_001753], tag TCCTGTAAAG, has a significant expression level in HGMEC (197 copies/200,000). Interestingly, the pathway analysis in HGMEC and dermal human microvascular EC (Vascular_normal_CS_control) SAGE libraries revealed novel differences regarding caveolae between these two microvascular endothelial cell types. In caveolae pathway caveolin-1, −2 and −3 were all present in HGMEC and absent in dermal microvascular EC. A complete list of molecules from this pathway present in HGMEC and absent in dermal microvascular EC is shown in Additional file 7: Table S5.

**Table 3:** **Excerpt of gene ontology (GO) cluster classification**

GO ID	P-value	HGMEC count	Total count	GO Term
**Cluster 1 (Translation and energy metabolism)**				
GO:0031145	2.64E-004	7	59	Anaphase-promoting complex-dependent proteasomal ubiquitin-dependent protein catabolic process
GO:0043161	2.24E-004	8	76	Proteasomal ubiquitin-dependent protein catabolic process
GO:0006511	3.08E-003	10	165	Ubiquitin-dependent protein catabolic process
GO:0044265	8.83E-004	15	274	Cellular macromolecule catabolic process
GO:0044260	2.60E-009	81	2044	Cellular macromolecule metabolic process
GO:0043170	1.84E-003	112	4217	Macromolecule metabolic process
GO:0006414	9.54E-049	41	78	Translational elongation
GO:0006412	2.67E-027	48	326	Translation
GO:0043284	3.35E-004	61	1871	Biopolymer biosynthetic process
GO:0009059	1.31E-004	72	2251	Macromolecule biosynthetic process
GO:0044249	2.86E-004	68	2142	Cellular biosynthetic process
GO:0009058	1.95E-003	76	2627	Biosynthetic process
GO:0044237	5.08E-003	128	5073	Cellular metabolic process
GO:0010467	1.39E-004	74	2337	Gene expression
GO:0009987	2.55E-004	184	7730	Cellular process
GO:0006120	3.76E-003	4	30	Mitochondrial electron transport, NADH to ubiquinone
GO:0042775	1.27E-004	6	37	Organelle ATP synthesis coupled electron transport
GO:0006119	1.90E-004	7	56	Oxidative phosphorylation
GO:0006096	9.71E-003	4	39	Glycolysis
GO:0006091	5.24E-005	14	187	Generation of precursor metabolites and energy
**Cluster 2 (Ubiquitination)**				
GO:0051437	4.62E-005	8	61	Positive regulation of ubiquitin-protein ligase activity during mitotic cell cycle
GO:0043085	2.93E-003	13	249	Positive regulation of catalytic activity
GO:0051436	2.37E-004	7	58	Negative regulation of ubiquitin-protein ligase activity during mitotic cell cycle
GO:0043086	8.56E-003	8	134	Negative regulation of catalytic activity
**Cluster 3 (Interspecies interaction)**				
GO:0044419	1.18E-004	13	177	Interspecies interaction between organisms
GO:0051704	5.88E-004	17	321	Multi-organism process
**Cluster 4 (Apoptosis)**				
GO:0006916	1.15E-003	9	120	Anti-apoptosis
GO:0043066	4.15E-003	10	172	Negative regulation of apoptosis
GO:0051093	6.73E-003	12	244	Negative regulation of developmental process
GO:0042981	4.69E-003	17	390	Regulation of apoptosis
GO:0006915	6.48E-003	22	575	Apoptosis
GO:0050793	7.01E-003	22	579	Regulation of developmental process
**Cluster 5 (Cytoskeleton organization)**				
GO:0030036	2.25E-003	10	158	Actin cytoskeleton organization and biogenesis
GO:0030029	1.32E-003	11	173	Actin filament-based process
GO:0007010	6.17E-003	16	368	Cytoskeleton organization and biogenesis

Into this GO analysis only 268 tags were included which were expressed in *ex vivo* glomeruli as well predominant to HGMEC when compared to other 16 types of EC. **Total count:** number of molecules present in the cluster; **HGMEC count:** how many of the total number of molecules in a cluster are present in HGMEC; **p-value:** comparison between the total number of members in a cluster and the count of those which are expressed in HGMEC as a sign of enrichment.

### Glomerular and non-glomerular endothelial expression of neurogranin

**Figure 5:**
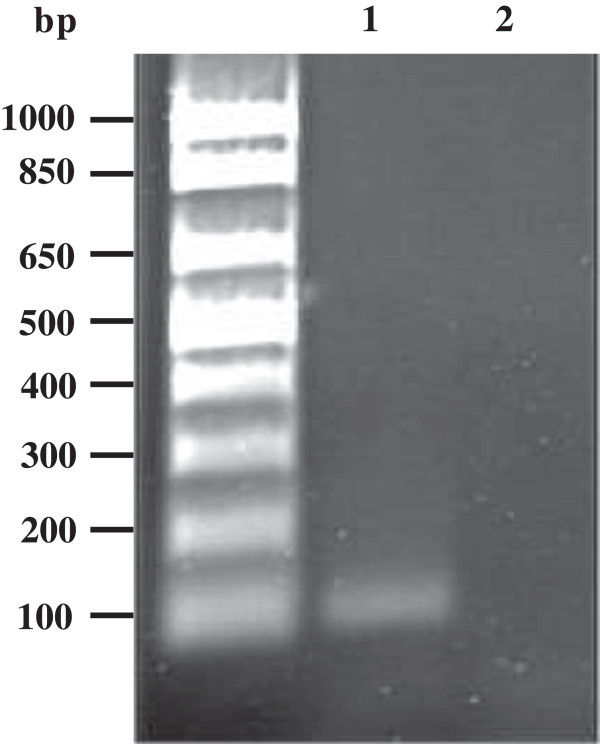
**Amplification of a 116 bp fragment of the human neurogranin cDNA from cDNA isolated from immortalized human glomerular endothelial cell line GEnC.** Agarose gel (1.5%) electrophoresis of 10 μL qPCR product using a template of cDNA generated by reverse transcription of total RNA isolated from GEnC (lane 1) or ddH_2_O as a negative control (lane 2).

**Figure 6:**
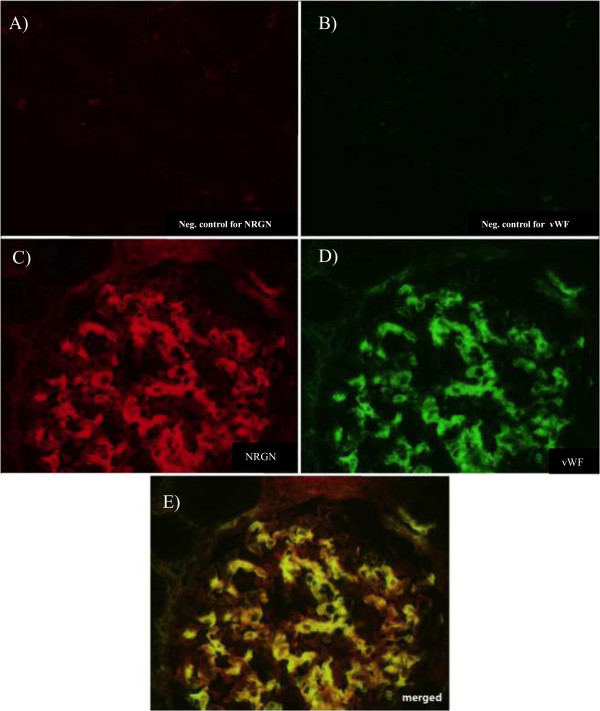
**Immunofluorescence images of sections of human kidney showing glomerular expression of NRGN.** Cryosections of human kidney were stained for neurogranin (NRGN) with anti-NRGN antibodies **(C)**, von-Willebrand factor (vWF) with anti-vWF antibodies **(D)** and second antibodies alone **(A** and **B)**. Signals of NRGN (red) and vWF (green) were merged in **(E)**.

To identify transcripts with the highest potential to be novel and HGMEC pre-dominant we applied the final group of transcripts of interest containing 268 tags (common to *ex vivo* glomeruli and differentially overexpressed in HGMEC when compared with non-glomerular endothelial cells) to a set of additional criteria: a) completely absent in all other nephron regions *ex vivo* [17] and b) more highly expressed than statistically expected in HGMEC. Ultimately, one transcript fulfilled all these criteria, namely NRGN. Its expression in HGMEC equalled to 192% of the statistically expected expression and it was enriched in HGMEC when compared to *ex vivo* glomerulus or to the fusion endothelial reference library with 52 versus 25 copies or 52 versus 21 copies per 200,000 tags, respectively. It was on position 174 when sorted from highest to the lowest expression level in the group of 268 tags (Additional file 4: Table S4). The corresponding tag for NRGN is TGACTGTGCT. Based on the SageGenie algorithm from Boon et al. [13] this tag has a high ranking of 95% and is classified as a reliable 3’ end of NRGN RefSeq transcripts [NM_001126181] and [NM_006176], which strongly supported the validity of the tag. Neurogranin transcript expression in GEnC under non-permissive conditions (5 days at 37°C) was shown by quantitative RT-PCR and the 116 bp long product was cloned, sequenced and matched the NRGN RefSeq transcript [NM_001126181], 237 bp, 100% (Figure 5). To confirm the expression of NRGN in the glomerular EC *in vivo*, frozen sections of human kidney were co-immunostained for NRGN (Figure 6C) and the EC marker vWF (Figure 6D). As shown in Figure 6E, both proteins show a significant co-localization confirming the transcriptional data that NRGN is expressed in the kidney’s glomerular EC. No reactivity was observed with secondary antibodies alone (Figure 6A and B). The endothelial expression of NRGN is further corroborated by the *in silico* analysis using each of the 18 publicly available endothelial libraries considered for the reference endothelial library. We found that 14 of them expressed the NRGN tag to some extent (Table 4), with a mean number of NRGN tags per 200,000 of 27.7 (standard deviation = 20.9).

**Table 4:** **List of all 18 non-glomerular endothelial SAGE libraries analyzed. 16 were merged to create the non-glomerular endothelial reference SAGE library**

SAGE library	Number of tags	Gene expression omnibus (GEO) dataset number	Number of NRGN tag per 200,000
Vascular_endothelium_breast_carcinoma_associated_P1H12 + _AP_DCIS6	65223	GSM384015	9
Vascular_endothelium_hemangioma_B_146	75680	GSM384016	8
Vascular_normal_CS_control	51562	GSM384019	0
Vascular_normal_CS_VEGF+	57316	GSM384020	7
Endothelium of normal colon	95327	provided by Dr. K. Kinzler	17
Endothelium of colon tumor	95543	provided by Dr. K. Kinzler	2
Microvascular endothelial cells cultured on advanced glycatedfibronectin and exposed to sustained high shear stress	24773	GSM45608	8
Microvascular endothelial cells cultured on fibronectin and exposed to sustained high shear stress	30615	GSM32266	19
Microvascular endothelial cells cultured on fibronectin and exposed to sustained low shear stress	31141	GSM41248	19
Vascular_endothelium_normal_liver_associated_AP_NLEC1	77759	GSM384018	0
Human Aortic Endothelial Cell Exposure to 0 h Short-Term Chronic Hypoxia (Control)	38446	GSM62240	57
Human Aortic Endothelial Cell Exposure to 8 h Short-Term Chronic Hypoxia	40629	GSM62241	39
Human Aortic Endothelial Cell Exposure to 24 h Short-Term Chronic Hypoxia	42371	GSM62242	52
Human Pulmonary Artery Endothelial Cell Exposure to 0 h Short-Term Chronic Hypoxia (Control)	25706	GSM62243	46
Human Pulmonary Artery Endothelial Cell Exposure to 8 h Short-Term Chronic Hypoxia	27666	GSM62244	58
Human Pulmonary Artery Endothelial Cell Exposure to 24 h Short-Term Chronic Hypoxia	42251	GSM62245	47
*Normal corneal endothelium*	*9537*	*GSM1652*	0
*Vascular_endothelium_normal breast_associated_P1H12 + _AP_1*	*34373*	GSM384017	0

The last two libraries in *italic* were not included in the non-glomerular endothelial reference SAGE library based on their failure to be clearly classified of endothelial origin according to our verification analysis as explained in Methods.

NRGN**:** neurogranin.

Two of the four libraries which did not contain the NRGN tag, were the ones which were below the “endothelial” threshold (Figure 3B) and thus not included into the fusion endothelial reference library. The other two were “Vascular normal_CS_control” from dermal microvascular EC and “Vascular_endothelium_normal_liver_associated_AP_NLEC1”. According to these data treatment of dermal microvascular EC with vascular growth factor (VEGF) known as a differentiation factor for EC [21] led to a detectable expression of NRGN, as shown in the “Vascular normal_CS_VEGF+” library (Table 4).

## Discussion

Human glomerular endothelium is composed of highly specialized cells. Their molecular features leading to the unique phenotype remain largely unknown. In this study we define the transcriptome of HGMEC using SAGE [12]. Serial analysis of gene expression gives exceptionally unbiased transcriptional data because it is based on extraction of unique sequence tags for each distinct transcript, known or unknown, differentially expressed or not, from a population of transcripts. The frequency with which a given tag is present in a SAGE library reflects the abundance of its transcript resulting in quantification of obtained data. Thus, it does not involve error prone statistics resulting from comparison to laboratory controls and thus its data are readily comparable among different laboratories. Moreover, it obtains a broader snapshot of gene expression when compared to the commercial arrays [22]. These clearly distinguish SAGE from other methods used for similar purposes. Also, in endothelial cell research SAGE has proven to be both reliable and successful [23–25]. It has to be noted that more recent and advanced high throughput sequencing methods such as deep RNA sequencing were considered for this study. This particular method displays similarities to SAGE regarding the linearity over a wide range of transcripts from low to high abundance as well as some technical advantages. It can work with significantly less starting material at a higher speed, with less work-load and cost for large sets of raw data from a single experiment to detect novel splice variants or alternative transcription sites [26, 27]. Yet, SAGE was the method of choice when designing the study due to the combination of its validity and the availability of unprecedented, large, well-maintained datasets and quality-controlled databases from SAGE libraries (e.g. Cancer Genome Anatomy Project including many endothelial cell types and glomeruli).

The main challenges of transcriptional studies remain a) the sequencing errors, which was reported to account for approximately 10% of tags in SAGE [28], b) the validation of detected transcripts, c) managing the vast number of transcripts and d) not “overseeing” novel transcripts of interest which are present, but not easily “visible” in the transcript library. In this study, these issues were addressed by incorporation of advanced bioinformatics and conventional methods such as real-time PCR and immunohistochemistry. Our bioinformatics strategy allowed verification of endothelial origin of the HGMEC as well as 16 out of the 18 SAGE libraries which were publicly available at the time (Table 4). The failure of two established endothelial SAGE libraries to qualify as endothelial using our methodology deserves to be mentioned. Although many possible explanations can be given for this phenomenon, we suggest that this is based on the significant differences between endothelia accounting for endothelial diversity. Nevertheless, we chose not to incorporate the transcript tags from these libraries into the non-glomerular endothelial reference library, although according to our calculations this would not change any statement or conclusion of this study. Furthermore, exclusion of singletons and comparison of HGMEC SAGE library first with *ex vivo* glomerular then with 16 endothelial SAGE libraries were fundamental. Only HGMEC-enriched tags with a significant overlap between HGMEC and *ex vivo* glomeruli under the simultaneous exclusion of common transcripts with non-glomerular endothelial cells were used for analyses, thus the probability of chasing tags based on sequencing errors was further minimized. Thus, expression of all the 20 randomly chosen transcripts in the resulting list of overexpressed HGMEC transcripts could be verified by qRT-PCR. Finally, robustness of our strategy was additionally validated by the demonstration of NRGN, a low-abundant molecule previously thought to be brain specific, in human glomerular endothelium *in vitro* and *in vivo* for the first time. Interestingly, although this transcript had never been known in kidney or any endothelial cell, our analysis showed that it was present in some previously generated endothelial SAGE libraries but was never recognized due to a lack of a strategy such as the one described in this study. The results from this stepwise procedure provide valuable large lists of transcripts expressed in human glomerular endothelium which have a high potential to be present *in vivo* and underline endothelial diversity.

Moreover, a genetic analysis of glomerular EC is especially intriguing: Not only because their uniqueness is evident in their morphology, behaviour in culture, and disease [2, 29–37], but also because it is postulated that they have a distinct embryological origin and that the developing kidney generates its own endothelium [38]. Because the origin of distinct HGMEC features *in vitro* or *in vivo* is not fully understood, we defined the transcriptome of human glomerular endothelium by means of SAGE using HGMEC in culture which carried typical characteristics of differentiated glomerular endothelium including fenestrations and particularly focused on transcripts which are common to HGMEC and non-cultured *ex vivo* glomeruli. Only those transcripts were exposed to further analysis regarding their presence or absence in a fusion endothelial reference library. Thus, the vast numbers of transcripts were gradually reduced to manageable numbers and to relevant lists which are presented here. Next, these lists were explored in increasing depth. At first glance the remarkable abundance of ribosomal proteins in the HGMEC SAGE library was evident by the presence of 23 different ribosomal proteins among the most abundant 50 tags. We argue that this is not a mere consequence of cell culture and is a pattern found in other SAGE libraries from non-cultured cells, e.g. human monocytes (27 ribosomal proteins in the most abundant 50 transcripts) [39]. High translational activity possessed by HGMEC was reflected by the overexpression of “translation and energy metabolism” GO cluster. According to the GO analysis another cluster which was more abundant than statistically expected in HGMEC was “ubiquitination”. Ubiquitination is a posttranslational modification of proteins. Monoubiquitination is involved in various processes e.g. in endocytosis or transcriptional regulation. In contrast, polyubiquitination of a protein results in recognition and degradation of this protein [40]. In this context, it is noteworthy that internalization and transport of proteins, particularly albumin, is of special interest in physiology as well as pathology of glomerular endothelium, both of which involve caveolins. A major caveolin is caveolin-1 (CAV1) which is significantly related to albumin excretion [41]. Ubiquitination seems to be responsible also for the turnover for CAV1. Hence, the presence of CAV1 in HGMEC SAGE library and enrichment of ubiquitination GO terms in addition to high concentration of ribosomal proteins substantiate the previously suggested characteristics of glomerular endothelium. Novel differences between different microvascular EC appeared by comparing caveolae pathway: in contrast to HGMEC the dermal microvascular EC (Vascular_normal_CS_control) did not express any of the caveolin 1–3 in addition to some other qualitative differences (Additional file 7: Table S5). Our results regarding the absence of ADAMTS13 in HGMEC are conflicting. The presence of this von Willebrand cleaving protease had been demonstrated in immortalized glomerular as well as in dermal microvascular EC before [42]. In most of the previously established endothelial SAGE libraries including dermal microvascular EC (Vascular_normal_CS_control and Vascular_normal_CS_VEGF) this molecule was not detected. Neither a complete kidney (SAGE_Kidney_normal_B_1, GSM383901, data not shown) nor the liver endothelial cell SAGE library (Vascular_endothelium_normal_liver_associated_AP_NLEC1, GSM384018, Table 4) expressed any ADAMTS13 tags, although liver and kidney cells were used as positive controls in the study by Tati et al. Only the SAGE library from endothelium of normal colon expressed ADAMTS13. Probably, this is due to the low abundance of this molecule as it was demonstrated by its appearance only at high cycle numbers of RT-PCR experiments especially in human glomerular and microvascular EC [42]. It can be argued that in spite of remarkable tag numbers the sizes of the SAGE libraries were too small to detect this transcript.

Finally, when obtained transcript lists were further filtered using additional criteria (expression in HGMEC higher then statistically expected, overexpressed in HGMEC when compared to the non-glomerular endothelial reference SAGE library, never before shown in kidney or any endothelial cell type, higher expression in HGMEC than in other endothelial cell types) only one low abundance transcript, namely NRGN, stood out. It is reasonable to assume that a larger size of HGMEC SAGE library would probably lead to the detection of a higher number of NRGN tags and more transcripts fulfilling same criteria. Subsequently, we first confirmed the presence of NRGN transcript in HGMEC by sequencing and then the *in vivo* expression of NRGN protein in human glomerular endothelium by immunohistochemistry. Thus far, NRGN was believed to be brain specific and abundant in forebrain neurons whose interactions with calmodulin are suggested to play an important role in the regulation of synaptic responses and plasticity [43, 44]. In brain, NRGN expression is dependent on thyroid hormones [45] and it is a direct target of thyroid hormone action [46–48]. There are numerous studies suggesting a link between thyroid and glomerular functions starting from embryogenesis [49] and involving chronic kidney disease as well as acute kidney injury [50], glomerular filtration rate [51] or resistant proteinuria [52]. Recent data in a rat model showed that proteinuria seen in hyperthyroidism is not due podocyte pathology and that hyper- as well as hypothyroidism lead to an increased capillary density when compared to control animals while hyperthyroidism resulted in an expansion of glomerular area [53]. Yet, molecules linking both organ systems have never been defined. We postulate that NRGN in glomerular endothelium represents a potential link between the kidney and the thyroid as this was described for brain and thyroid. Studies are underway to test the function of glomerular endothelial NRGN in the thyroid-kidney axis.

## Conclusions

In this study we describe and analyze a HGMEC SAGE library as the first quantitative description of the human glomerular endothelial transcriptome. By using open-source SAGE data from uncultured glomeruli and from 16 other non-glomerular endothelial cell types which were merged to gain a reference endothelial library as well as by employing specific *in-silico* analyses an efficient research strategy was established. In this method we included an analysis to verify the declared origin of large transcript lists and strongly suggest this or similar analyses should be employed whenever using such transcript lists before including them in the studies, because 11% of the analyzed endothelial SAGE libraries failed to qualify as such. Also, integration of additional stringent filters into the bioinformatics to reduce the vast number of transcripts to manageable groups appeared to be indispensable. The described multi-step strategy which also involved GO and pathway analyses was capable of determining a list of genes potentially expressed *in vivo*, highlighting glomerular endothelial uniqueness as well as endothelial diversity and identifying the low abundant NRGN for the first time in glomerular endothelium and confirming its expression *in vitro* and *in vivo.* The demonstration of NRGN as a molecule potentially linking glomerulus to thyroid and the sufficiency of our transcriptomic data providing a novel insight in significant differences between the glomerular and other endothelia represent novel and exciting findings in glomerular endothelial research.

## Methods

### Culture of primary HGMEC

Human glomerular microvascular EC were isolated and cultured as previously described [54]. Briefly, human renal tissue was obtained, with informed consent, from macroscopically healthy pieces of nephrectomized kidneys. Written consent from patients was obtained before the surgery (Ethics Committee of the Medical University of Vienna protocol approval number EK 141/2002 047/05/2008 ensuring adherence to The Declaration of Helsinki). Isolated glomeruli from minced cortex were digested and plated on fibronectin coated cell culture dishes. After 7 days in cell culture medium including fetal calf serum (FCS), 50 μg/mL endothelial cell growth supplement, 30 U/mL heparin, 100 U/mL penicillin, 100 μg/mL streptomycin, and 2 mM alanine-glutamine HGMEC constituted approx. 10% of glomerular outgrowths. HGMEC were isolated from this mixed culture using CD31 immuno-magnetic sorting as per manufacturer’s instructions (Dynal, Hamburg, Germany). HGMEC were passaged 4 to 8 times.

### Immortalized human glomerular endothelial cell line

Conditionally immortalized GEnC line was kindly provided by Dr. Satchell and Dr. Mathieson (University of Bristol, UK) and these cells were kept as instructed [9].

### HGMEC characterisation

HGMEC were seeded onto fibronectin coated glass cover slips, and cultured until near confluence prior to fixation. For immunostaining of intracellular antigens cell monolayers were permeabilised by incubation in 1% Triton-X-100 for 20 min. Non-specific binding sites were blocked by incubating 1 h in blocking solution (5% (w/v) non-fat milk in phosphate-buffered saline (PBS). Primary antibody was incubated at 1 μg/mL for CD62P/E and CD31 and at 30 μg/mL for vWF for 1 hour. After probes were rinsed 3-times Texas red conjugated secondary antibody was incubated at 1:200 in antibody solution. To test the upregulation of CD62P/E cells were treated with human recombinant TNF alpha for 12 hours at 100U/mL.

### Transmission electron microscopy of HGMEC

Human glomerular EC grown on microporous inserts were fixed with 1% glutaraldehyde, buffered with PBS, post fixed in 1% OsO_4_ buffered with Na-cacodylate (0.1 M), dehydrated in graded series of ethanol, and embedded in Polybed. Ultrathin sections were stained conventionally by uranyl acetate and lead citrate and were examined with a JOEL 100 C electron microscope at 100 KV.

### Scanning electron microscopy of HGMEC

HGMEC were fixed with Karnowsky’s fixative, post fixed in 1% OsO_4_ buffered with Na-cacodylate (0.1 M), dehydrated in graded series of methanol, and submitted to critical point drying using CO_2_. Dried specimen were sputter coated with a 120 Å gold palladium layer and examined with a JOEL JSM 25-S scanning electron microscope.

### Construction of the HGMEC SAGE library

SAGE libraries were constructed from cultured primary HGMEC according to the short SAGE protocol [12] as described at http://www.sagenet.org with modifications described previously [23, 24].

### Confirmation of the endothelial origin of the HGMEC SAGE library

To confirm the endothelial origin of HGMEC SAGE library we applied a novel classification strategy as a learning process to distinguish endothelial libraries from others. Positive examples were eighteen endothelial SAGE libraries (Table 4) not including HGMEC. Negative examples were 63 SAGE libraries from non-endothelial normal tissues obtained from SAGE Genie (Additional file 8: Table S6). Only tag counts ≥2 were used and all tags which occurred in at least one of the libraries with a count ≥2 were considered. These were included in a tag-wise Wilcoxon rank sum test with the null hypothesis that the median expression of each tag is equal in both classes, and the alternative hypothesis that the median expression is larger in the endothelial libraries compared to the other libraries. The strategy was to select *n* tags highly expressed in endothelial libraries. In a subsequent step, the sum of the relative copy number of these tags was defined as an indicator for endothelial origin. The threshold was defined as the cut-off value to minimize wrong classifications based on the histogram consisting of sum of copy numbers of *n* tags. As shown in Figure 3B the chosen threshold 0.022 resulted in only 2 wrong classifications. To determine the optimal number of tags (*n*) to best distinguish endothelial from non-endothelial, we repeated the above described procedure using different numbers of *n* from 50 to 1000 tags (Figure 3A). The minimum number of false classifications was observed for n = 100 or 150 (Figure 3A). We chose 150 tags as depicted in Additional file 2: Table S2 for classifying the HGMEC SAGE library. Using these tags and the threshold of 0.022 the HGMEC SAGE library was clearly classified as endothelial (Figure 3B).

### Construction of a non-glomerular endothelial reference SAGE library

To obtain an endothelial reference library, sixteen endothelial SAGE libraries (Table 4) were merged to form a cumulative endothelial SAGE library as a reference library consisting of 822,008 tags.

### Analysis of differential expression of transcripts and tag-to-gene mapping

First, all tags with a copy number = 1 were eliminated from the HGMEC SAGE library. For determining the transcripts potentially present *in vivo* and specific for HGMEC we then combined the information present in the HGMEC SAGE library and in the *ex vivo* glomerular library by Chabardes-Garonne et al. [17]. We designated a transcript as being a potential HGMEC transcript if it occurred at least three times in the HGMEC SAGE library and in the *ex vivo* glomerular library*.* Each of these HGMEC transcripts was tested for overexpression compared with the cumulative non-glomerular endothelial reference SAGE library (Chi-Square-Test, p < 0.01).

Comparisons between libraries (HGMEC, glomerular, cumulative non-glomerular endothelial reference SAGE library) were performed in a Microsoft ACCESS database. Resulting tags (common or differentially expressed among libraries) or entire tag lists from libraries were automatically identified over the web by means of “IdenTAG”. IdenTAG was written in PERL specifically for this study and implemented a web-client tailored for the SAGE Genie site *(*http://cgap.nci.nih.gov/SAGE/AnatomicViewer*)* [13] of the Cancer Genome Anatomy Project at the US National Cancer Institute.

### Gene Ontology and pathway analysis

For GO analysis of the overexpressed transcripts the UniGene-IDs were mapped via SAGE Genie to the corresponding Gene Symbols. Overrepresentation of genes in a GO category was determined by the Fisher test (p < 0.01). Subsequently, we identified the main functional categories present in these overexpressed genes by mapping them to GO Biological Process and determining whether they occur more often in a category than expected. All statistical analyses including the false discovery rate correction (p < 0.05) and the annotation with GO Terms were performed using R (http://www.r-project.org) and Bioconductor (http://www.bioconductor.org). For pathway analysis the software PathwayArchitect from Stratagene (La Jolla, CA, USA) was used.

### Quantitative real time PCR

An ABI Prism 7700 Sequence Detector (Applied Biosystems, California, USA) was used for mRNA quantification via real-time PCR. Complementary DNA from HGMEC, GEnC cultured at 37°C or from human brain was generated. For human NRGN (assay ID details: Hs00183469) GEnC cDNA was used. Confirmation RT-PCR reactions for the 20 randomly selected transcripts were performed using HGMEC cDNA (List of transcripts and assay ID in Table 2). Beta-actin was used as endogenous control gene (Applied Biosystems, Hs99999903_m1). Normalization of Ct values of each gene (ß-actin and NRGN) and determination of NRGN expression in GEnC cells grown at 37°C was calculated according to the 2 ^ΔΔCt^ method [55].

### Selection, cloning and sequencing of NRGN

We searched for novel transcripts with the highest potential to be expressed in HGMEC *in vivo* based on 4 criteria: 1) common to *ex vivo* glomerulum (which consequently contains non-cultured glomerular endothelial cells) and HGMEC SAGE library; 2) complete absence in the remaining seven nephron segment SAGE libraries namely proximal convoluted tubule, proximal straight tubule, medullary thick ascending limb of Henle’s loop, cortical thick ascending limb of Henle’s loop, cortical collecting duct, outer medullary collecting duct and distal convoluted tubule containing approximately 350,000 tags [17]; 3) never before shown in EC, 4) enrichment of the transcript in HGMEC: observed expression higher in HGMEC when compared to the non-glomerular endothelial reference library and to the statistically expected count in HGMEC. After NRGN was recognized as the only tag fulfilling these criteria its qRT-PCR product (116 bp) was purified and cloned in a pDrive cloning vector. Minipreps were prepared using the Qiaprep spin Miniprep Kit (Qiagen, Valencia, CA, USA) and vectors containing the insert were selected by EcoRI digestion and sequenced using the M13 reverse primer.

### Neurogranin immunofluorescence staining in human kidney sections

For immunofluorescence, 15 μm cryostat sections of human kidney tissue from the fully anonymized archive at the Department of Pathology of the Medical University of Vienna ensuring compliance to the guidelines from the Medical University of Vienna and adherence to The Declaration of Helsinki (see section “Culture of primary HGMEC” above) were fixated and blocked. They were then incubated with 1:500 rabbit anti- NRGN polyclonal or anti-vWF monoclonal antibody for 1 hour. Primary antibodies were visualized using the TSA Plus Kit (PerkinElmer, Wellesley, MA, USA) and photographed with a fluorescence microscope.

## Electronic supplementary material

Additional file 1: Table S1: Complete HGMEC SAGE library after exclusion of linker sequences. (XLS 4 MB)

Additional file 2: Table S2: List of 150 tags used for endothelial classification as described in Figure 3. (XLS 34 KB)

Additional file 3: Table S3: Complete list of 823 tags common to SAGE libraries from HGMEC and *ex vivo* glomeruli. FDR: multiple test correction by false discovery rate. (XLS 159 KB)

Additional file 4: Table S4: Complete list of 268 tags which are common to SAGE libraries from HGMEC and *ex vivo* glomeruli and overexpressed in HGMEC compared to the 16 non-glomerular endothelial cell SAGE libraries including information on cytogenetic location of each tag. FDR: multiple test correction by false discovery rate. (XLS 94 KB)

Additional file 5: Figure S1: Volcano plot showing p-values against log fold change for the 823 transcripts from Additional file 3: Table S3. Each red dot symbolizes one transcript which is common to HGMEC and *ex vivo* glomeruli and is enriched in HGMEC when compared to non-glomerular endothelial cells (268 transcripts from Additional file 4: Table S4). The plot shows a typical appearance except that because of the high p-values some of the sample points are accumulated on both over- (positive values on the x-axis) and underrepresentation (negative values on the x-axis) sides. Note the logarithmic scaling of the y-axis. (PDF 15 KB)

Additional file 6: Figure S2: Chromosome distribution of 268 HGMEC enriched genes as listed in Additional file 4: Table S4. (DOC 33 KB)

Additional file 7: Table S5: Lists of caveolae pathway molecules present in HGMEC and absent in dermal microvascular endothelial cells. Connectivity: high degree of connectivity represents proteins which are more likely to be essential for survival. (XLS 25 KB)

Additional file 8: Table S6: List of the 63 non-endothelial SAGE libraries which were used for the algorithm to verify the endothelial origin of a given SAGE library. (XLS 28 KB)

## Abbreviations

Bp: Base pair
CAV1: Caveolin-1
EC: Endothelial cell
EM: Electron microscopy
GEnC: Glomerular endothelial cell
GEO: Gene expression omnibus
GO: Gene ontology
HGMEC: Human glomerular microvascular endothelial cells
NRGN: Neurogranin
PBS: Phosphate-buffered saline
PCR: Polymerase chain reaction
PECAM1: Platelet/endothelial cell adhesion molecule 1
RT-PCR: Real time polymerase chain reaction
SAGE: Serial analysis of gene expression
SEM: Scanning electron microscopy
TNF: Tumor necrosis factor
VEGF: Vascular growth factor
vWF: Von Willebrand factor.
