# Supplementary figures and images for: A SAGE based approach to human glomerular endothelium: defining the transcriptome, finding a novel molecule and highlighting endothelial diversity
Source: BMC Genomics. 2014 Aug 27;15(1):725. doi: 10.1186/1471-2164-15-725 (PMC4156628; doi:10.1186/1471-2164-15-725)

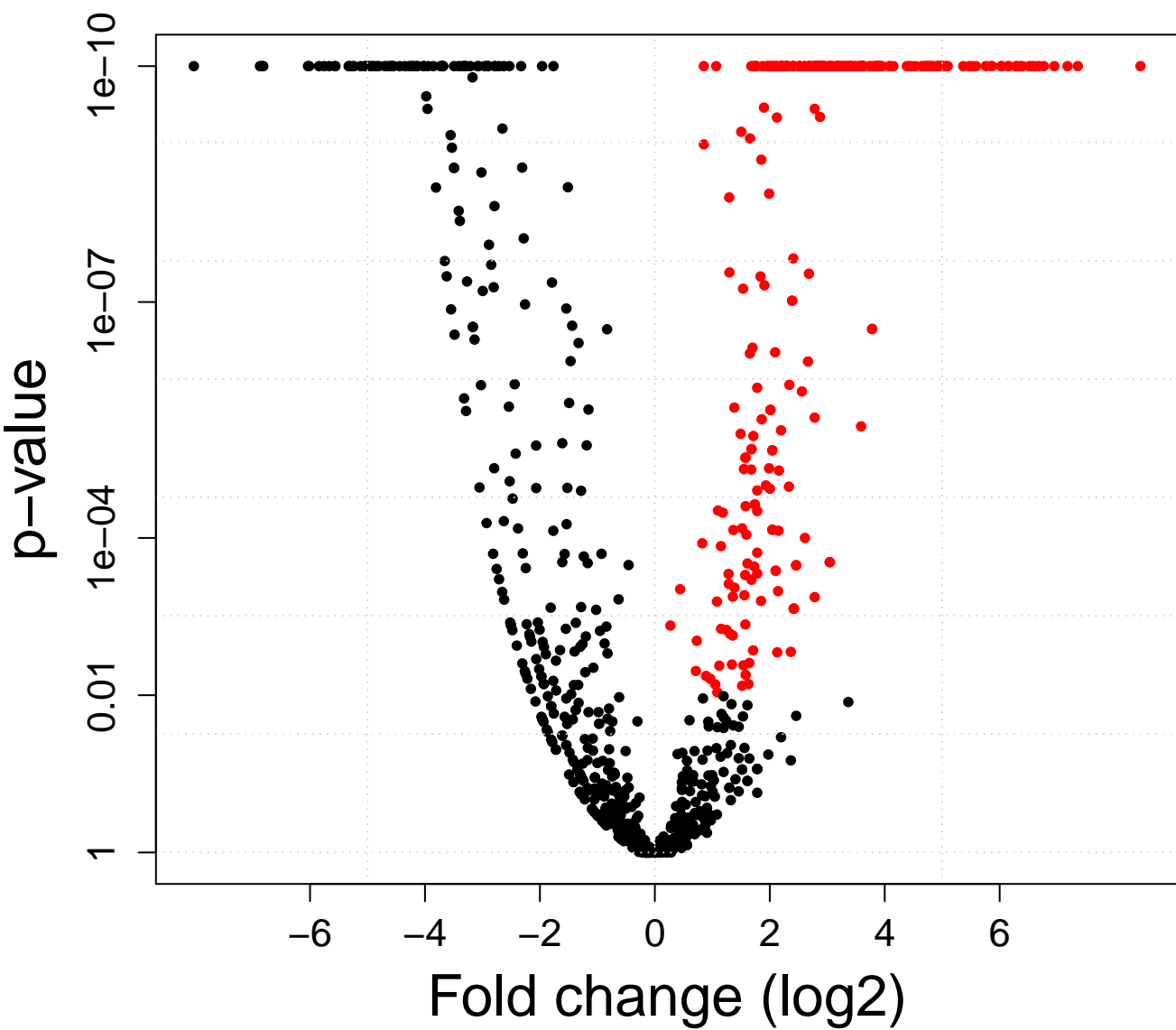

Supplement: Supplementary file 5 — Additional file 5: Figure S1: Volcano plot showing p-values against log fold change for the 823 transcripts from Additional file 3: Table S3. Each red dot symbolizes one transcript which is common to HGMEC and ex vivo glomeruli and is enriched in HGMEC when compared to non-glomerular endothelial cells (268 transcripts from Additional file 4: Table S4). The plot shows a typical appearance except that because of the high p-values some of the sample points are accumulated on both over- (positive values on the x-axis) and underrepresentation (negative values on the x-axis) sides. Note the logarithmic scaling of the y-axis. (PDF 15 KB) [file 12864_2014_6406_MOESM5_ESM.pdf]
