# Supplementary material for: A SAGE based approach to human glomerular endothelium: defining the transcriptome, finding a novel molecule and highlighting endothelial diversity
Source: BMC Genomics. 2014 Aug 27;15(1):725. doi: 10.1186/1471-2164-15-725 (PMC4156628; doi:10.1186/1471-2164-15-725)
Supplement: Supplementary file 6 — Additional file 6: Figure S2: Chromosome distribution of 268 HGMEC enriched genes as listed in Additional file 4: Table S4. (DOC 33 KB) [file 12864_2014_6406_MOESM6_ESM.doc]

**Additional file 6: Figure S2**
